# PRMT5 disruption drives antitumor immunity in cervical cancer by reprogramming T cell-mediated response and regulating PD-L1 expression

**DOI:** 10.7150/thno.59605

**Published:** 2021-08-28

**Authors:** Yongshuai Jiang, Yuanyang Yuan, Ming Chen, Shengzhe Li, Jun Bai, Yuanteng Zhang, Ying Sun, Guojue Wang, Haiyan Xu, Ziyu Wang, Yingxia Zheng, Hong Nie

**Affiliations:** 1 Shanghai Institute of Immunology, Department of Immunology and Microbiology, Shanghai Jiao Tong University School of Medicine, Shanghai 200025, China.; 2 Department of Gynecology, Shanghai Ninth People's Hospital, Shanghai Jiao Tong University School of Medicine, Shanghai 200011, China.; 3 Department of Laboratory Medicine, Xin Hua Hospital, Shanghai Jiao Tong University School of Medicine, Shanghai 200092, China.

**Keywords:** PRMT5, PD-L1, STAT1, cervical cancer, tumor microenvironment

## Abstract

**Rationale**: Protein arginine methyltransferase 5 (PRMT5) is an oncogene that promotes tumor cell proliferation, invasion and metastasis. However, the underlying mechanisms by which PRMT5 contributes to the progression of cervical cancer and especially the tumor microenvironment remain poorly understood.

**Methods**: PRMT5 expression level was analyzed by Q-PCR, western blot, immunohistochemistry, and TCGA database. The role of PRMT5 in tumor growth was observed by transplanted tumor models, and the function of T cells in tumor microenvironment and in vitro co-culture system was investigated through flow cytometry. The transcriptional regulation of PRMT5 was analyzed using luciferase reporter and chromatin immunoprecipitation (ChIP) assay. The therapeutic effect of PRMT5 inhibitor was evaluated in a cervical cancer cell line transplanted tumor model.

**Results**: We observed that the mRNA and protein expression levels of PRMT5 were increased in cervical cancer tissues, and the high expression of PRMT5 was associated with poor outcomes in cervical cancer patients. The absence of PRMT5 significantly inhibited tumor growth in a cervical cancer transplanted tumor model, and importantly, PRMT5 absence in tumors led to increase the number and enhance the function of tumor infiltrating T cells. Mechanistically, PRMT5 enhanced the transcription of STAT1 through symmetric dimethylation of histone H3R2 and thus promoted PD-L1 expression in cervical cancer cells. Moreover, in an in vitro co-culture system, knockdown of PRMT5 in tumor cells could directly enhance the expression of IFN-γ, TNF-α and granzyme B in T cells. These results suggested that PRMT5 promoted the development of cervical cancer by the crosstalk between tumor cells and T cells. Furthermore, the PRMT5 inhibitor EPZ015666 treatment could suppress tumor growth in a cervical cancer transplanted tumor model.

**Conclusion**: Our results clarify a new mechanism which PRMT5 knockdown in cervical cancer cells drives an antitumor function via reprogramming T cell-mediated response and regulating PD-L1 expression. Thus, our study highlights that PRMT5 may be a potential target for cervical cancer therapy.

## Introduction

Cervical cancer is the most common malignancy that affects women's life span, especially in developing countries. It has been reported cervical cancer causes more than 300 000 deaths worldwide each year 1. Cervical cancer is mainly caused by high-risk human papilloma virus (hrHPV). Although HPV vaccine can prevent the occurrence of cervical cancer, it has almost no function for those who have been infected with HPV, and this population is still likely to develop cervical cancer 2-4. The overall prognosis remains poor for those patients who have metastatic or recurrent cervical cancer 5. As the molecular mechanism of cervical cancer is not yet fully understood, there is limited success in improving the disease free survival rate of cervical cancer patients. Therefore, we need to further explore the mechanism of cervical cancer and find new targets for its clinical treatment.

Methylation of arginine residues is one of the most prevalent post-translational modifications (PTMs). Arginine methylation is an important regulator of biological function and impacts oncogenesis and tumor progression 6. Protein arginine methyltransferase can be classified into three types: monomethylarginine (MMA), symmetric dimethylarginine (SDMA) and asymmetric dimethylarginine (ADMA), with potential different functional consequences for each type of methylation. Protein arginine methyltransferase 5 (PRMT5), which is intimately linked to carcinogenesis, belongs to the type II enzyme class that catalyzes symmetric dimethylarginine of histone and nonhistone proteins 7. PRMT5 can activate or repress gene expression by generating histone marks, such as H3R2me2s, H3R8me2s, or H4R3me2s 8-13. PRMT5 has emerged as a pivotal molecule in tumorigenesis and metastasis 14-17. However, its underlying mechanism has not been fully investigated.

Programmed death-1 (PD-1) is highly expressed on activated T cells. Its ligand, programmed death-ligand 1 (PD-L1, also known as CD274), is mainly expressed on tumor cells in the tumor microenvironment 18. It is well known that PD-L1 binds to PD-1 on T cells, inhibiting T cell function. The activation of the PD-1/PD-L1 signaling pathway can cause tyrosine residue phosphorylation in the ITSM structure domain of PD-1 cytoplasmic portion. PD-1/PD-L1 activation can decrease the antitumor activity of tumor infiltrating lymphocytes (TILs) by inducing apoptosis, inhibiting the production of granular enzyme/perforin, and decreasing the secretion of IFN-γ/IL-2/TNF-α 19. Currently, there are several known stimulators of PD-L1 expression, including IFN-γ, TNF-α, cell growth factors, hypoxia, and exosomes 20, 21. IFN-γ mediates JAK/STAT1 pathway and upregulates PD-L1 gene transcription 22, 23.

The cellular constituents of the tumor microenvironment not only include vascular endothelial cells, tumor cells, stromal cells and extracellular matrix (ECM), but also contain many immune cells, and studies have provided clear evidence that infiltrating T cells, natural killer (NK) cells, macrophages, B cells and dendritic cells (DCs) play an important role in tumor progression 24-26. Few reports have been published about how PRMT5 affects immune cells in the tumor microenvironment. PRMT5 is an important modulator of CD4^+^ T cell expansion and IL-2 production 27. T cell-specific deletion of PRMT5 was shown to lead to a marked reduction in thymic iNKT cells, as well as a decreased number of peripheral CD4^+^ T cells and CD8^+^ T cells 28. Conditional knockout (cKO) of PRMT5 in Treg cells results in severe autoimmune disease, and Treg number and function are limited 29.

Here, we found that in cervical cancer, PRMT5 expression was correlated well with the poor prognosis of disease. Further, we explored the molecular mechanism of PRMT5 promoting the expression of PD-L1 in cervical cancer cells, and studied the effect of PRMT5 on the number and function of T cells in the tumor microenvironment of a cervical cancer transplanted tumor model. We illuminated the mechanism of PRMT5 in the development of cervical cancer and explored the feasibility of treating cervical cancer with PRMT5 as a target.

## Results

### Abnormal accumulation of PRMT5 was observed in cervical cancer

**Figure 1:**
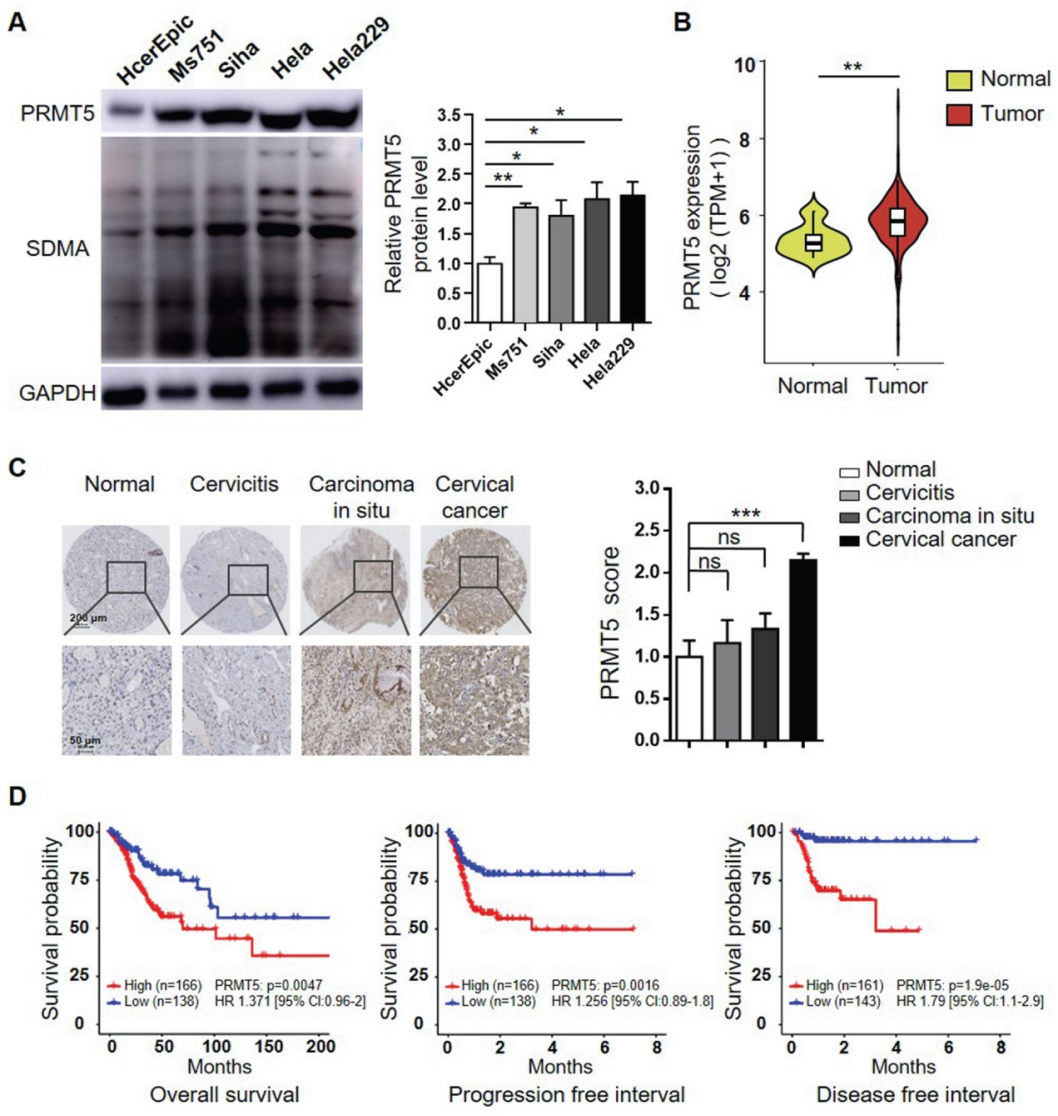
** Increases of PRMT5 expression correlated with poor prognosis in cervical cancer.** (**A**) PRMT5 expression and SDMA levels in a normal human cervical cell line and four human cervical cancer cell lines were analyzed using western blot (left panel), and the protein expression of PRMT5 was quantified by ImageJ (right panel). Values are presented as the mean ± SEM. (**B**) Violin plot showed PRMT5 expression of normal cervical tissues (n = 13) and cervical tumor tissues (n = 306) from TCGA database (Kruskal-Wallis test). (**C**) PRMT5 expression levels of tissue array from normal cervix (n = 4), cervicitis (n = 3), carcinoma in situ (n = 3) and cervical cancer (n = 35) were analyzed using immunohistochemistry method. Values are presented as the mean ± SEM. (**D**) OS (left panel), PFI (middle panel) and DFI (right panel) of cervical cancer patients with different PRMT5 levels were analyzed from TCGA database. The "maxstat.test" function in R package maxstat was used to dichotomy gene expression, and all potential cutting points were repeatedly tested to find the maximum rank statistic, and then the patients were divided into the PRMT5-high group and the PRMT5-low group according to the maximum selected log-rank statistics, so as to reduce the calculated batch effect. Survival curves were generated using the Kaplan-Meier method, and the log-rank test was used to determine the significance of the differences. Univariate Cox regression model was used to calculate the hazard ratio (HR). *P < 0.05, **P < 0.01, and ***P < 0.001.

To investigate the potential oncogenic role of PRMT5 and whether it could be a target for treating cervical cancer, we initially assessed the PRMT5 protein levels in one normal human cervical cell line and four human cervical cancer cell lines. As shown in Figure 1A, PRMT5 expression and SDMA level were significantly higher in cancer cells than in normal cells. We further used data obtained from TCGA cohort to analyze the expression levels of PRMT5 in normal cervical tissues and tumor tissues. The result showed that cervical tumor tissues expressed higher levels of PRMT5 compared to normal tissues (Figure 1B). Next, we analyzed PRMT5 expression in cervical tissues from cervical cancer patients and controls using immunohistochemistry (IHC). From tissue microarray, we found that PRMT5 expression in cervical cancer tissues was higher than that in normal cervix specimens (Figure 1C). Moreover, upon TCGA database, cervical cancer patients with high PRMT5 expression were found to have a significantly worse overall survival (OS), progression free interval (PFI) and disease free interval (DFI) (Figure 1D). In addition, Cox proportional hazards regression analysis showed that PRMT5 high expression was a risk factor for incidence of cervical cancer. Overall, these data suggested that PRMT5 expression was increased in cervical cancer and that increased PRMT5 expression was associated with poor prognosis.

### Downregulation of PRMT5 attenuated tumor growth in an immunocompetent mouse cervical cancer model

**Figure 2:**
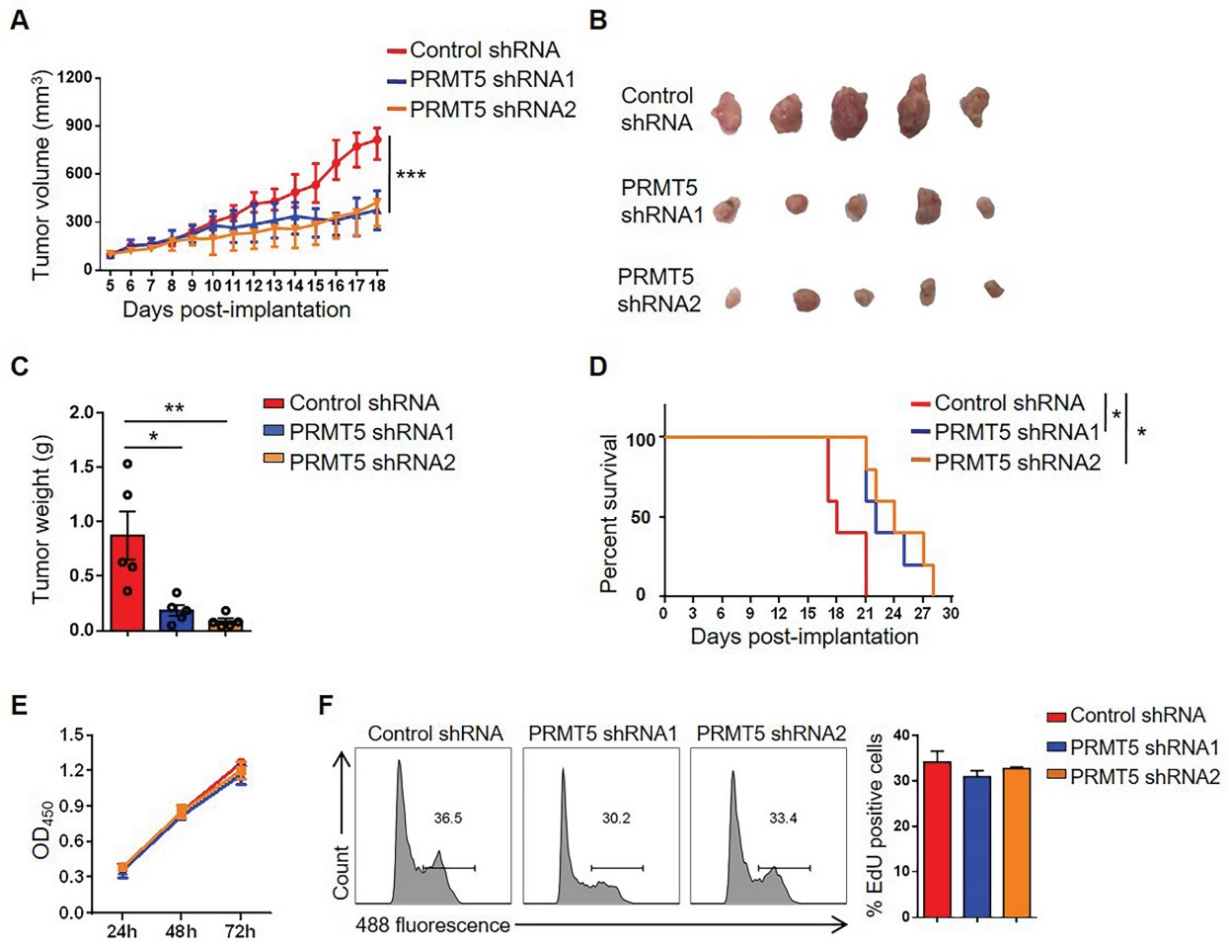
** Downregulation of PRMT5 inhibited tumor growth.** (**A-C**) Control cells and PRMT5 knockdown U14 cells were subcutaneously injected into 6-week-old female C57BL/6 mice (n = 5 for each group). (**A**) A line graph shows the tumor growth curve of mice. Images (**B**) and weight (**C**) of the resected tumor at day 18 after inoculation. Data are representative of at least two independent experiments. Values are presented as the mean ± SEM. (**D**) Survival curve of tumor-bearing mice subcutaneously injected with control cells and PRMT5 knockdown U14 cells (n = 5 for each group) (log-rank test). (**E-F**) Proliferation of control cells and PRMT5 knockdown U14 cells were analyzed by CCK-8 assay (**E**) and EdU staining (**F**). Data are representative of at least two independent experiments. Values are presented as the mean ± SEM. *P < 0.05, **P < 0.01, and ***P < 0.001.

To further determine the oncogenic properties of PRMT5 in cervical cancer, stable PRMT5 knockdown U14 cell lines were established. We found PRMT5 expression and SDMA level were decreased in PRMT5 knockdown cells (Figure S1A). To study the effect of PRMT5 in tumorigenesis of cervical cancer, we established tumor models in C57BL/6 mice with control cells and PRMT5 knockdown U14 cells. The results showed that both the volumes and weights of the tumors in the PRMT5 knockdown groups were significantly decreased (Figure 2A-C) and conferred a marked survival benefit when compared with those of the control group (Figure 2D), demonstrating that the lack of PRMT5 suppressed the tumorigenicity of cervical cancer in vivo.

It was reported PRMT5 had an effect on tumor growth 30, we wondered whether tumor cell proliferation changed the tumor size. Therefore, we tested the effect of PRMT5 knockdown on tumor cell proliferation with CCK8 assay and EdU staining. We found that knockdown of PRMT5 had no effect on the proliferation of U14 cells in vitro (Figure 2E-F). Subsequently, we randomly selected one of the stable PRMT5 knockdown U14 cell lines and subcutaneously injected the cells into nude mice. However, there was no obvious effect on the tumor volume, tumor weight or survival rate (Figure S1B-E), and these data suggested that PRMT5 had no effect on the proliferation of U14 cells in immune-deficient mice. Thus, we considered that the immune system might play a crucial role in the development of a transplanted tumor model. Taken together, these data revealed that downregulation of PRMT5 was conducive to tumor inhibition.

### PRMT5 deficiency in tumor cells induced host antitumor immunity

**Figure 3:**
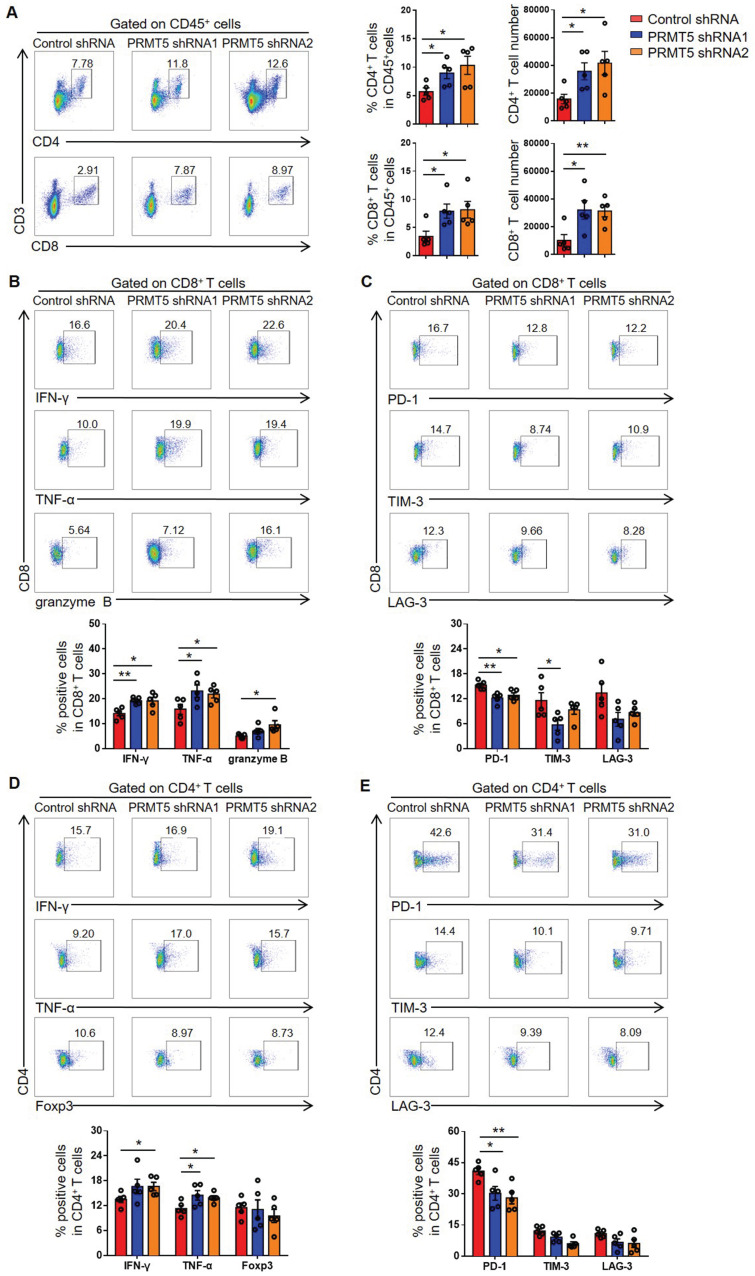
** PRMT5 deficiency in tumor cells affected tumor infiltrating T cells.** Control cells and PRMT5 knockdown U14 cells were subcutaneously injected into 6-week-old female C57BL/6 mice (n = 5 for each group). Mice were euthanized at day 8 after inoculation. The tumor single cell suspension was prepared and analyzed by flow cytometry. (**A**) The percentage of CD4^+^ and CD8^+^ T cells in CD45^+^ cells, and the absolute number of the two T cell subsets in tumors. (**B**) Expression of IFN-γ, TNF-α and granzyme B in CD8^+^ T cells. (**C**) Expression of PD-1, TIM-3 and LAG-3 on the surface of CD8^+^ T cells. (**D**) Expression of IFN-γ, TNF-α and Foxp3 in CD4^+^ T cells. (**E**) Expression of PD-1, TIM-3 and LAG-3 on the surface of CD4^+^ T cells. Values are presented as the mean ± SEM. *P < 0.05 and **P < 0.01.

Various tumor infiltrating cells, including T cells, macrophages, DCs, B cells and NK cells, have been shown to play important roles in the tumor microenvironment*.* To ascertain whether PRMT5 knockdown promoted an antitumor immunity, we performed flow cytometry analyses on these cell populations and found that the percentage of CD3^+^ T cells was significantly increased and the percentage of macrophages was notably decreased in the PRMT5 knockdown tumor microenvironment, while the percentages of other cells (such as DCs, B cells and NK cells) showed no statistical differences (Figure S2A). As we all know, T cells play an important role in antitumor immunity, we further investigated the effect of T cells on tumor growth in vivo and how PRMT5 affected the function of T cells in the tumor microenvironment. Compared to control group, the percentage and the number of CD4^+^ T and CD8^+^ T cells in PRMT5 knockdown tumor microenvironment were significantly higher (Figure 3A). However, PRMT5 deficiency in tumor cells had no effect on the proliferation of T cells in the tumor microenvironment (Figure S2B).

Furthermore, the function of T cells was tested through flow cytometry, and we found that PRMT5 deficiency in tumor cells enhanced IFN-γ, TNF-α and granzyme B secretion by CD8^+^ T cells (Figure 3B). Notably, along with PRMT5 deficiency in tumor cells, the expression of PD-1 and TIM-3 on CD8^+^ T cells was decreased and LAG-3 expression had a declined trend (Figure 3C). We also found that PRMT5 deficiency in tumor cells enhanced the secretion of the cytokines IFN-γ and TNF-α by CD4^+^ T cells (Figure 3D), and the expression pattern of above checkpoints on CD4^+^ T cells was similar to that on CD8^+^ T cells (Figure 3E). These findings indicated that PRMT5 had a tumor intrinsic effect that limited T cell infiltration and altered the expression of cytokines and coinhibitory molecules of T cells in cervical cancer. In vitro, the results of a co-culture experiment of tumor cells and T cells showed that the knockdown of PRMT5 in tumor cells could enhance the expression of IFN-γ, TNF-α and granzyme B in T cells (Figure S3A), and overexpression of PRMT5 in tumor cells could decrease the expression of these cytokines in T cells (Figure S3B). The above results suggested that the knockdown of PRMT5 in tumor cells could play an antitumor function by increasing the number and enhancing the function of tumor infiltrating T cells.

### PRMT5 regulated PD-L1 expression through the IFNγ/JAK/STAT1 axis

**Figure 4:**
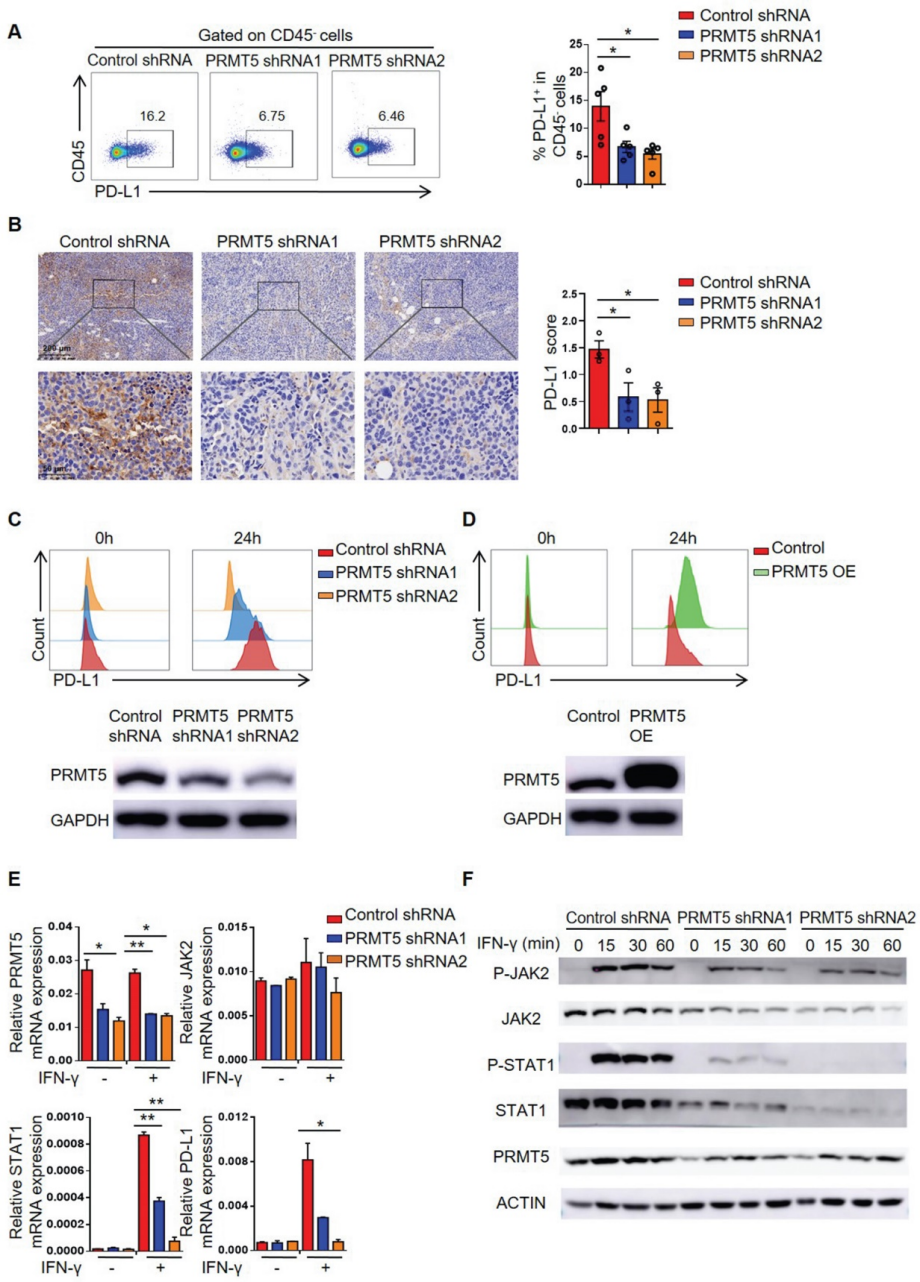
** PRMT5 promoted STAT1 and PD-L1 expression.** (**A-B**) Control cells and PRMT5 knockdown U14 cells were subcutaneously injected into 6-week-old female C57BL/6 mice. Mice were euthanized at day 8 after inoculation. (**A**) The tumor single cell suspension was prepared and the percentage of PD-L1 expression on CD45^-^ cells was analyzed by flow cytometry (n = 5 for each group). (**B**) Immunohistochemical staining and statistical analysis of PD-L1 in tumor tissues (n = 3 for each group). (**C-E**) Control cells, PRMT5 knockdown U14 cells and PRMT5-overexpressing U14 cells were stimulated with IFN-γ for 24 h. PD-L1 expression on PRMT5 knockdown U14 cells (**C**) and PRMT5-overexpressing U14 cells (**D**) was analyzed using flow cytometry. (**E**) Real-time PCR experiments were used to test the expression of the indicated genes. (**F**) Western blot experiments were used to test the expression of the indicated proteins after IFN-γ stimulation for different time. Data are representative of at least two independent experiments (**C-F**). Values are presented as the mean ± SEM. *P < 0.05 and **P < 0.01.

The PD-1/PD-L1 pathway generates inhibitory signals in the tumor microenvironment, so we further tested the expression of PD-L1 on tumor cells. Interestingly, we found that PRMT5 deficiency in tumor cells decreased PD-L1 expression (Figure 4A-B), which could promote antitumor immunity. IFN-γ was reported to induce the expression of PD-L1 in tumors 31. Therefore, we examined whether PRMT5 alteration in U14 cells regulated PD-L1 expression in response to IFN-γ stimulation. Compared with the control group, after stimulation with IFN-γ, PD-L1 expression was decreased in U14 cells with PRMT5 knockdown, whereas PD-L1 expression was upregulated in the PRMT5-overexpressing U14 cells (Figure 4C-D). These data suggested that PD-L1 expression was positively correlated with PRMT5. Based on these results, we wanted to further elucidate the mechanism by which PRMT5 regulated PD-L1 expression. It was reported IFN-γ could upregulate PD-L1 expression through the JAK/STAT1 pathway 22, 32. Therefore, we analyzed the expression of PD-L1 and related genes after stimulation with IFN-γ. We found that knockdown of PRMT5 reduced STAT1 and PD-L1 expression at the RNA level and protein level (Figure 4C, E-F). Taken together, these data indicated that PRMT5 might regulate the expression of STAT1 and PD-L1 through the IFNγ/JAK/STAT1 pathway.

### PRMT5 regulated STAT1 gene transcription through symmetric dimethylation of histone H3R2

**Figure 5:**
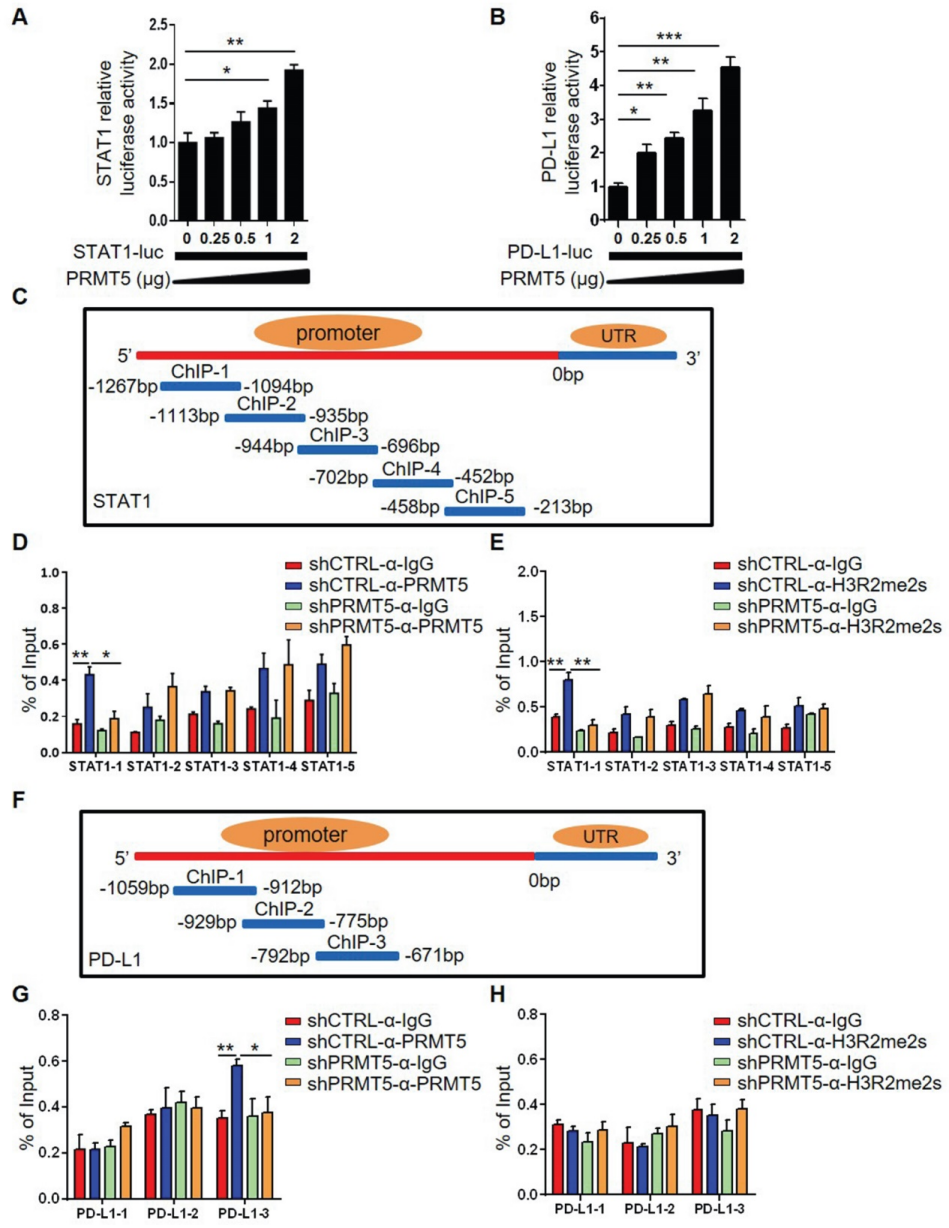
** PRMT5 regulated STAT1 transcription through H3R2me2s.** Effect of PRMT5 on STAT1 (**A**) and PD-L1 (**B**) transcription was analyzed through dual-luciferase reporter assays. (**C**) The primer design scheme for the STAT1 promoter and its fragment in the ChIP assay. (**D-E**) Enrichment of PRMT5, H3R2me2s or IgG at the STAT1 promoter was assessed by ChIP Q-PCR. (**F**) The primer design scheme for the PD-L1 promoter and its fragment in the ChIP assay. (**G-H**) Enrichment of PRMT5, H3R2me2s or IgG at the PD-L1 promoter was assessed by ChIP Q-PCR. Data are representative of at least two independent experiments. Values are presented as the mean ± SEM. *P < 0.05, **P < 0.01, and *** P < 0.001.

To clarify whether PRMT5 directly regulated STAT1 and PD-L1 transcription, we constructed plasmids bearing STAT1 and PD-L1 promoter regions. A luciferase reporter assay showed that PRMT5 promoted STAT1 and PD-L1 transcription (Figure 5A-B). To further confirm whether PRMT5 binds directly to the promoter region of STAT1 and PD-L1, we designed ChIP PCR primers for STAT1 and PD-L1 promoter regions, with each fragment being about 300-400bp. ChIP PCR assays showed that PRMT5 was able to bind to the promoters of STAT1 in the -1269bp to -212bp and PD-L1 in the -1060bp to -679bp (Figure S4A-B). PRMT5 can activate or inhibit gene expression by dimethylating the arginine residues of histones, and one mechanism by which PRMT5 promotes gene expression is through the symmetric methylation of H3R2, H3R8 and H4R3 8-12. Our results showed that PRMT5 indeed increased the expression of symmetric dimethylation of histone H3R2, H3R8 and H4R3 (Figure S4C). In order to further narrow down the binding range of PRMT5 to STAT1 and PD-L1 promoter regions, five and three pairs of ChIP Q-PCR primers that corresponded to the STAT1 and PD-L1 promoter were designed, and each spanned an approximately 200 bp fragment within the promoter (Figure 5C, 5F). Our data showed that PRMT5 enhanced STAT1 transcription by binding to the ChIP-1 promoter region between -1267 bp and -1094 bp. Importantly, PRMT5 deletion significantly reduced STAT1 promoter occupancy (Figure 5D), confirming that PRMT5 is truly recruited to the STAT1 promoter. Consistent with this chain of events, H3R2me2s was enriched at this STAT1 promoter in a PRMT5-dependent manner (Figure 5E), while the histone H3R8me2s and H4R3me2s were not enriched at STAT1 promoter region (Figure S4D-E). The experimental results also showed that PRMT5 enhanced PD-L1 transcription by binding to the ChIP-3 promoter region between -792 bp and -671 bp (Figure 5G), but it could not affect the transcription of PD-L1 gene by methylating histone H3R2, H3R8 and H4R3 (Figure 5H, Figure S4F-G). In addition, a STAT1 inhibitor (Fludarabine) blocked IFN-γ-induced PD-L1 expression in cervical cancer cells (Figure S5), further confirming that PRMT5 regulated PD-L1 expression through STAT1. Collectively, these findings suggested that STAT1 expression affected by PRMT5-mediated epigenetic regulation could drive PD-L1 expression, which promoted the development of cervical cancer.

### The PRMT5 inhibitor EPZ015666 exhibited a therapeutic effect on cervical cancer mice model

**Figure 6:**
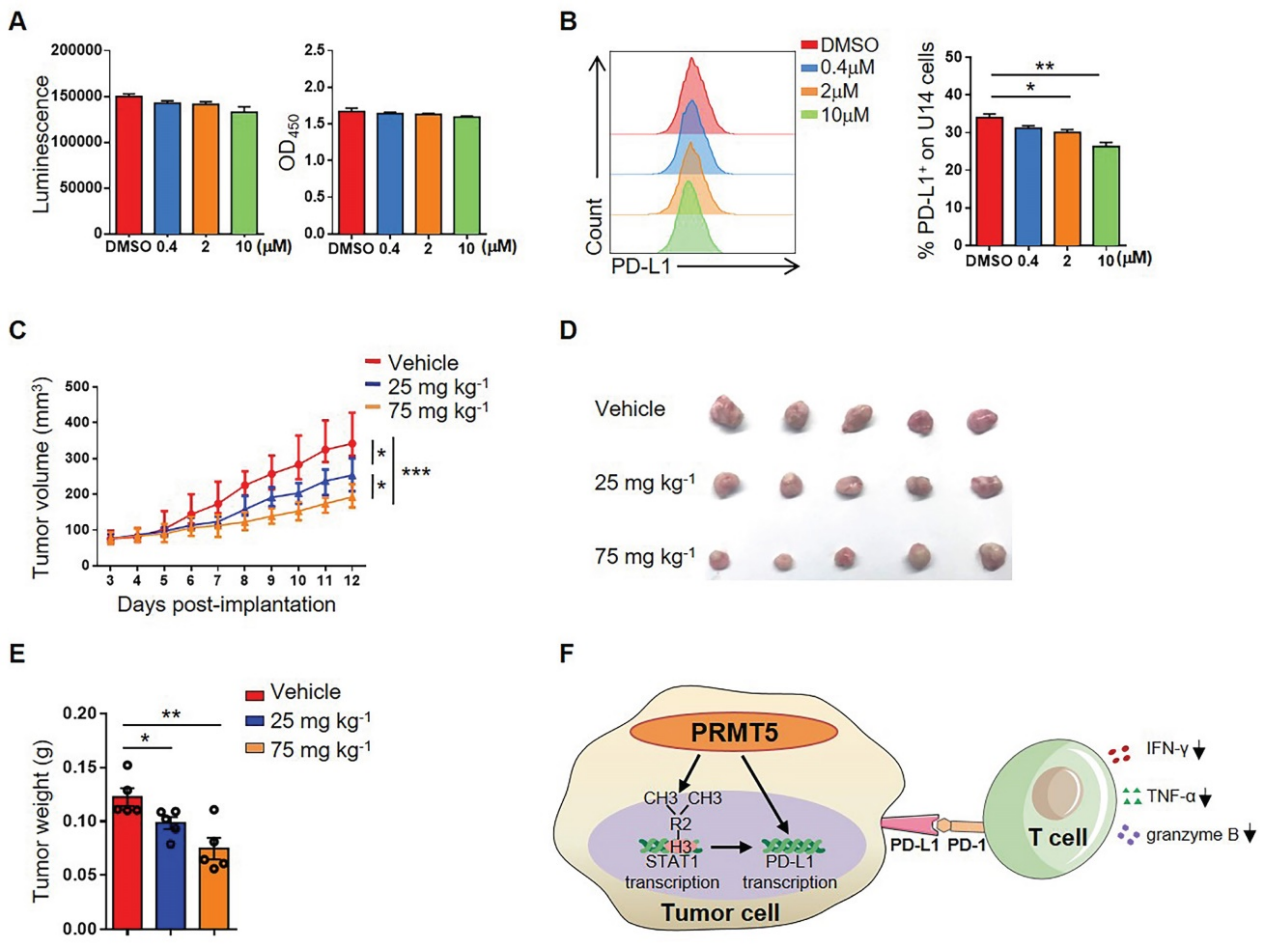
** PRMT5 inhibitor EPZ015666 suppressed cervical cancer growth.** (**A-B**) U14 cells were cultured in the indicated concentrations of EPZ015666 for 72 h. (**A**) Effect of EPZ015666 on the viability and proliferation of U14 cells was analyzed by CellTiter-Glo luminescent assay and CCK-8 Cell Counting assay, respectively. (**B**) Expression of PD-L1 on U14 cells was analyzed by flow cytometry after IFN-γ stimulation. (**C-E**) On day 3 after inoculation of U14 cells, EPZ015666 was intraperitoneally injected into 6-week-old female C57BL/6 mice every day (n = 5 for each group). (**C**) A line graph shows the tumor growth curve of mice. Images (**D**) and weight (**E**) of the resected tumor at day 12 after inoculation. Data are representative of two independent experiments. Values are presented as the mean ± SEM. (**F**) Schematic diagram of the mechanism of PRMT5 in promoting the development of cervical cancer. *P < 0.05, **P < 0.01, and *** P < 0.001.

To further explore whether PRMT5 could be an effective target for the treatment of cervical cancer, we selected EPZ015666, a specific inhibitor of PRMT5, to assess a possible therapeutic effect. We found that there was no effect on the viability and proliferation of U14 cells when treated with EPZ015666 at concentrations of up to 10 µM in vitro (Figure 6A). Likewise, we discovered that EPZ015666 treatment also inhibited the expression of PD-L1 on U14 cells (Figure 6B). The results indicated that the phenotype of U14 cells treated with EPZ015666 is consistent with that of the PRMT5 knockdown U14 cells. Since PRMT5 inhibitors not only have effects on tumor cells, but also affect the function of immune cells. Therefore, we also observed the effect of EPZ015666 on immune cells in vitro. The results showed that there was no effect on the viability and proliferation of primary mouse spleen cells when treated with EPZ015666 at concentrations of up to 10 µM (Figure S6A). Besides, flow cytometry results showed that EPZ015666 treatment had no differences in CD4^+^ and CD8^+^ T cell frequency (Figure S6B), but increased the cytokine secretion from CD4^+^ and CD8^+^ T cells, such as IFN-γ, TNF-α and granzyme B (Figure S6C-D). Therefore, we observed the effect of EPZ015666 therapy on cervical cancer mice model and found that EPZ015666 treatment reduced the tumor volume, tumor size and tumor weight in mice, and this therapeutic effect was dose-dependent (Figure 6C-E). These findings suggested that PRMT5 could be an attractive therapeutic target for the treatment of cervical cancer.

## Discussion

PRMT5 is known as an oncogene in various carcinomas, but at present the mechanism of PRMT5 has not been fully understood in cervical cancer, especially in the aspect of the crosstalk between tumor cells and T cells in the tumor microenvironment. In this study, we firstly uncovered that PRMT5 knockdown in cervical cancer cells decreased PD-L1 expression by regulating the transcription of STAT1 gene through symmetric dimethylation of histone H3R2, and increased the number and function of T cells in the tumor microenvironment. Our results clarified a new mechanism which PRMT5 knockdown in cervical cancer cells drives an antitumor function via reprogramming T cell-mediated response and regulating PD-L1 expression, and indicated PRMT5 inhibitor could be a potential therapeutic strategy in cervical cancer.

High-risk human papilloma virus infection is the major cause of cervical cancer. The mechanism is that E6 and E7 two early virus genes inactivate the function of two tumor suppressor proteins, p53 and Rb, thus promote cervical carcinogenesis. Arginine methylation and protein synthesis of P53 were regulated by PRMT5 33. It has been reported that PRMT5 restricts Hepatitis B virus (HBV) replication through repression of HBV DNA transcription 34, but the relationship between PRMT5 and HPV infection were not known. PRMT5 functions as a key regulator of cancer development. In breast cancer and lung cancer, PRMT5 promotes tumor cell proliferation by regulating the cell cycle 35, 36. However, in some kinds of cancer cells, such as B16 cells, PRMT5 expression is not correlated with cell proliferation 37. Likewise, we found that PRMT5 expression did not affect the proliferation of a cervical cancer cell line in vitro. One possible explanation is that the methylation target of PRMT5 in different cancers may not be the same.

In this study, we discovered that PRMT5 deletion in tumor cells inhibited the tumor growth in a cervical cancer model of C57BL/6 mice, not in a model of nude mice, clearly implying that the immune cells in the tumor microenvironment indeed played an important role. Our results also revealed that PRMT5 deficiency in tumor cells increased the percentage and the number of CD4^+^ T and CD8^+^ T cells, which is consistent with an earlier report of the role of PRMT5 in melanoma 37. Furthermore, we found that PRMT5 deficiency in tumor cells had no effect on the proliferation of T cells in the tumor microenvironment. Given the importance of T cells in the tumor microenvironment, we tested cytokine secretion in tumor infiltrating CD4^+^ T cells and CD8^+^ T cells. We found that in PRMT5 knockdown tumor microenvironment, the expression of IFN-γ and TNF-α in CD4^+^ T and CD8^+^ T cells were increased, and the expression of granzyme B in CD8^+^ T cells was also upregulated. As reported, PD-1, TIM-3 and LAG-3 are important coinhibitory molecules. These coinhibitory molecules on T cells bind to the corresponding ligands on tumor cells and antigen-presenting cells to produce inhibitory signals to T cells. Interference with the PD-1/PD-L1 signaling pathway can remove the inhibition of T cells and restore their function. In PRMT5 knockdown tumor microenvironment, we found the expression of PD-1 on the surface of T cells was decreased and the function of T cells was restored. These observations suggested that PRMT5 deficiency in tumor cells enhanced host antitumor immunity.

Subsequently, we further investigated the mechanism of antitumor immunity caused by PRMT5 deficiency. IFN-γ activates the JAK/STAT1 pathway and induces the expression of classical interferon-stimulated genes (ISG) 23. Recruitment and phosphorylation of STAT1 can regulate transcription of interferon regulatory factor-1 (IRF-1) in the nucleus, and transcription of IRF-1 promotes PD-L1 expression 32. In this study, we found that PRMT5 was able to bind to both STAT1 promoter and PD-L1 promoter region. And it was reported that H3R2me2s, H3R8me2s, and H4R3me2s methylation are all regulated by PRMT5 8-12. We determined that PRMT5 could drive STAT1 transcription through symmetric dimethylation of histone H3R2, which then induced the expression of PD-L1. But we could not find PRMT5 regulated PD-L1 transcription by symmetric dimethylation of histones H3R2, H3R8 and H4R3. We also noticed STAT1 blocking reagent Fludarabine could prevent PD-L1 expression of cervical cancer cells induced by IFN-γ, thus proving PRMT5 did regulate PD-L1 expression depending on STAT1. The above results indicated two possibilities. One is that PRMT5 regulates STAT1 transcription and then STAT1 regulates PD-L1 transcription as we already know. The other is that PRMT5 can regulate PD-L1 transcription maybe through an unknown transcription factor which can bind to its promoter. Further studies are warranted to fully understand the mechanism. In brief, we unravel a new mechanism about how PRMT5 promotes tumor growth. In tumor cells, PRMT5 regulates the expression of STAT1 and PD-L1. And in tumor microenvironment, the interaction between PD-L1 and PD-1 can inhibit IFN-γ, TNF-α and granzyme B secretion in T cells (Figure 6F).

A selective inhibitor of PRMT5, EPZ015666 (also known as GSK3235025), was reported in the treatment of mantle cell lymphoma (MCL) model 38. In our study, EPZ015666 showed significant and dose-dependent antitumor activity in a cervical cancer transplanted tumor model. Besides, several PRMT5 inhibitors, including DS-437, LLY-283, CMP5, HLCL-61, C220 and PR5-LL-CM01, have been also used to treat different types of tumors, including human cancer cells 39. For T cells, a PRMT5 inhibitor C220 cause inhibition of mouse and human allogeneic T cell proliferation and IFN-γ and IL-17 cytokine production 40. And it has been reported that EPZ015666 treatment reduced human T cell proliferation, viability, and functionality 41. Until now, three PRMT5 inhibitors (GSK3326595, JNJ-64619178 and PF-06939999) are being used in six clinical trials (NCT02783300, NCT03573310, NCT03614728, NCT03854227, NCT04555473, NCT04676516) for the therapy of hematologic and solid tumors, containing primary and metastatic tumors. In METEOR-1 study, which investigate the safety, pharmacokinetics (PK), pharmacodynamics (PD), and efficacy of GSK3326595, the patients with advanced or metastatic solid tumors were responded to the therapy, and adverse event (AEs) were commonly happened but still manageable 42. JNJ-64619178 is orally active and has been found to inhibit the tumor growth in different cancer cell lines and in mouse xenograft models of human non-small cell lung cancer and small cell lung cancer 43. PF-06939999 is a PRMT5 inhibitor that potentially inhibits proliferative and neoplastic activities 44. Whether PRMT5 inhibitors are effective in cervical cancer patients requires further investigation. PRMT5 expression is upregulated in cervical cancer and can promote EMT procession, while high levels of PRMT8 expression were also observed in cervical cancer 45, 46. It has been reported that PRMT1 expression maybe a potential predictive marker for neoadjuvant chemotherapy treatment in patients with locally advanced cervical cancer 47. Combination of inhibitors of PRMT1 and PRMT5 has a synergistic cancer cell growth inhibition in pancreatic cancer and DLBCL cell lines and in pancreatic adenocarcinoma xenografts mouse models 48. In this study, we have not checked the expression and function of other PRMTs family members and whether these PRMTs have crosstalk or not still need to be further explored. In addition, it has been reported that methylthioadenosine phosphorylase (MTAP) cleaves MTA to generate precursor substrates for methionine and adenine salvage pathways. Deletion of MTAP often occurs in cancer, and results in accumulation of MTA 49. PRMT5 inhibition is potentially beneficial in MTAP loss patients as MTA only partially inhibits the methyltransferase activity of PRMT5, and whether it is overruled is depended on the dosage that used. However, this study at least provides a theoretical basis for the use of PRMT5 inhibitors in the treatment of cervical cancer.

Overall, our study elucidated a new mechanism which PRMT5 knockdown in cervical cancer cells drives an antitumor function via reprogramming T cell-mediated response and regulating PD-L1 expression. Importantly, we found that the elevated expression of PRMT5 was significantly correlated poor survival outcome of cervical cancer patients, and the PRMT5 inhibitor therapy was effective in a tumor-bearing mouse model of cervical cancer. These findings suggested that PRMT5 acted as an oncogenic driver in the development of cervical cancer, and could be an effective target for clinical treatment.

## Materials and Methods

### Cell lines

A normal human cervical cell line (HcerEpic), four human cervical cancer cell lines (Siha, Ms751, HeLa, and Hela229), and a mouse cervical cancer cell line (U14) were used in this study. HcerEpic was purchased from Shanghai Guan&Dao Biological Engineering Company, and U14 was purchased from the National Infrastructure of Cell Line Resource (Beijing). All cells were cultured at 37 °C in a humidified incubator with 5% CO_2_ in Dulbecco's modified Eagle's medium (DMEM) (HyClone) or Roswell Park Memorial Institute (RPMI) 1640 medium (Gibco) supplemented with 10% fetal bovine serum (FBS) (HyClone), 100 U/mL penicillin and 100 μg/mL streptomycin (Gibco).

### Cell transfection

PRMT5 knockdown was achieved using the short hairpin RNA (shRNA)-mediated stable silencing method. Two short hairpin RNAs targeting mouse PRMT5 were designed. shRNA1: AGCCAGGTGACAGTTGTCTCATCAGACAT and shRNA2: TTCCTGTGGAGGTGAACACGGTGCTTCAT. Two short hairpin RNAs targeting human PRMT5 were designed. shRNA1: GGCTCAAGCCACCAATCTATG and shRNA2: GCCCAGTTTGAGATGCCTTAT. 293T cells were seeded at 5×10^4^ cells per well in 24-well plates, and cell transfection was performed using Lipofectamine 2000 (Invitrogen). Viruses were produced by transfecting the 293T cells with control plasmid, PRMT5 knockdown plasmid and PRMT5-overexpressing plasmid. Viruses were collected for 48 h of transfection and were used to infect U14 cells or Siha cells. Then, PRMT5 knockdown cells, PRMT5-overexpressing cells, and their corresponding control cells were obtained after screening with 2 µg/mL puromycin (Sigma-Aldrich).

### IHC analysis

A tissue microarray (CXC962) was purchased from Shanghai Superbiotek Company. IHC was performed using an anti-PRMT5 antibody (1:50, #ab109451, Abcam). Each sample was assigned a score according to the intensity of the staining (0 = no staining, 1 = weak staining, 2 = moderate staining, and 3 = strong staining) and the proportion of the stained cells (1 = 1%-25%, 2 = 25%-50%, 3 = 50%-75%, and 4 = 75%-100%). The stained tissues were scored by clinical pathologists. Mouse IHC analysis was performed to determine PD-L1 expression in cervical cancer tissues using an anti-PD-L1 antibody (1:50, #ab213480, Abcam). The results were scanned with Digital Pathology Slide Scanner (KF-PRO-120).

### Animal experiments

Female C57BL/6 mice and nude mice were purchased from Shanghai Lingchang Biotechnology Company. All mice were maintained in a specific pathogen-free facility, and all animal experiments were performed in accordance with protocols approved by the Institutional Animal Care and Use Committee of Shanghai Jiao Tong University School of Medicine. Mice at 6 w of age were used for the experiments. Control cells and PRMT5 knockdown U14 cells in 100 μL (0.1×10^6^ cells) were subcutaneously implanted into the right flanks of nude mice. Control cells and PRMT5 knockdown U14 cells in 100 μL (3×10^6^ cells) were subcutaneously implanted into the right flanks of C57BL/6 mice. In vivo therapy assay, U14 cells in 100 μL (5×10^6^ cells) were subcutaneously implanted into the right flanks of C57BL/6 mice. At day 3 after inoculation of U14 cells, the PRMT5 inhibitor EPZ015666 was intraperitoneally injected every day. The tumor length (L) and width (W) were monitored by measurement with a digital caliper every day. Tumor volume was calculated with the formula: V=LW^2^/2, and tumor volume more than 800 mm^3^ was as the standard of mice death. Tumor-bearing mice were calculated survival rate and drawn survival curve.

### Flow cytometry

Single cell suspensions were harvested from fresh tissues according to standard procedures, and the cells were labeled in FACS buffer (PBS with 2% FBS and 1mM EDTA) with monoclonal antibodies against surface markers, which were obtained from eBioscience, BD Pharmingen and Biolegend Company. For intracellular cytokine staining, cells were stimulated with cell stimulation cocktail (plus protein transport inhibitors) (Thermo Fisher Scientific) for 5 h before surface staining. After surface staining, cells were fixed and permeabilized with BD Cytofix/Cytoperm buffer (BD Biosciences) or Foxp3 Transcription Factor Staining Buffer Set (eBioscience), following the manufacturer's instructions. Then, they were stained with antibodies against intracellular cytokines or Foxp3. Sample data were acquired on a BD LSRFortessa X20 (BD Biosciences) and analyzed with FlowJo software (TreeStar). Antibody information is listed in Table S1.

### Quantitative real-time PCR (Q-PCR)

Total RNA was extracted from cells following a standard TRIzol (Invitrogen) protocol. cDNA was synthesized from total RNA using PrimerScript^TM^ RT Reagent kit (Takara). Gene expression was detected using SYBR Green-based real-time PCR, which was performed with an ABI Step One Q-PCR Detection System (Life Technologies). Data were normalized to the mRNA levels of β-actin or GAPDH. The primers used are listed in Table S2.

### Western blot

Cells were harvested and lysed in 1× SDS loading buffer (Beyotime), and proteins were transferred onto nitrocellulose membranes (Millipore). The membranes were blocked with 5% nonfat milk and then were incubated with primary antibody overnight at 4°C on a rotator. Then, the membranes were incubated with a secondary antibody, and the bands were visualized using an ECL kit (Thermo Fisher Scientific). The following primary antibodies were used: PRMT5 (#ab109451, Abcam), JAK2 (#3230T, Cell Signaling Technology), P-JAK2 (#8082T, Cell Signaling Technology), STAT1 (#9172S, Cell Signaling Technology), P-STAT1 (#7649T, Cell Signaling Technology), ACTIN (#abs830031, Absin), GAPDH (#abs830030, Absin), H3 (#ab1791, Abcam), H3R2me2s (#ab194684, Abcam), H3R8me2s (#A-3706, EpiGentek), H4R3me2s (#ab5823, Abcam) and H4 (#2935S, Cell Signaling Technology).

### Co-culture experiment

Mouse spleen T cells sorted with CD90.2 Microbeads (Miltenyi Biotec) were co-cultured with PRMT5 knockdown U14 cells, PRMT5-overexpressing U14 cells or its corresponding control group cells at ratio of 10:1. Cells were stimulated with anti-CD3 (2 μg/mL) and anti-CD28 (2 μg/mL) for 48 h. Samples were analyzed by flow cytometry.

### Luciferase reporter assay

The promoter regions of STAT1 and PD-L1 were amplified from human DNA and then were cloned into a PGL4 vector (Promega). 293T cells were seeded in 24-well plates 24 h before transfection. The STAT1 or PD-L1 promoter reporter constructs were co-transfected along with PRMT5-overexpressing plasmid or a control vector using Lipofectamine 2000 (Invitrogen). After 48 h, luciferase activity was assessed using the Dual-Luciferase Reporter reagent (Promega) according to the manufacturer's protocol. Renilla luciferase was used for normalization.

### Chromatin immunoprecipitation (ChIP) assay

The ChIP assay was carried out according to the instruction of ChIP assay kit (#53008, Active Motif). Siha cells were stimulated with IFN-γ (40 ng/mL) for 24 h. Next, the cells were harvested and cross-linked in 1% formaldehyde for 10 min at room temperature. Then, the cells were treated with glycine and incubated for 5 min at room temperature. Cells were washed with ice-cold PBS and then were centrifuged. Then, we discarded the supernatant and resuspended the cells in lysis buffer supplemented with 5 μL of protease inhibitor cocktail (PIC) and 5 μL of PMSF. The samples were sonicated, and 50 μL of products were removed to assess the DNA fragment size. The remainder was stored at -80 °C. Antibodies against control IgG (#2729S, Cell Signaling Technology), PRMT5 (#61001, Active Motif), H3R2me2s (#ab194684, Abcam), H3R8me2s (#A-3706, EpiGentek) and H4R3me2s (#ab5823, Abcam) were used for the ChIP assay. 10 μL of immunoprecipitated sample was used as input control. Beads were washed with ChIP buffer I once and then ChIP buffer II twice. The immunoprecipitated chromatin was eluted with ChIP elution buffer and incubated at 37 °C for 15 min, which was followed by a 15 min incubation at 95 °C in a thermocycler to reverse the cross-links. Then, 2 μL of Proteinase K was added, and the samples were incubated at 37 °C for 60 min. Finally, 2 μL of Proteinase K stop solution was added. The bound DNA fragments were verified by PCR. In addition, DNA was amplified by quantitative real-time PCR and normalized to the input levels. Primer sequences for ChIP PCR are listed in Table S3, and primer sequences for ChIP Q-PCR are listed in Table S4.

### Cell viability and cell proliferation assay

U14 cells or spleen cells were seeded in 96-well plates at a density of 1×10^4^ cells or 5×10^5^ cells (100 μL/well) and incubated at 37 °C with 5% CO_2_. Medium with EPZ015666 at different concentrations was added to each well and incubated for 72 h. The viability of U14 cells or spleen cells was analyzed by Cell Titer-Glo luminescent assay (Promega). The luminesence values were measured with SpectraMax i3x (Molecular Devices). A CCK-8 Cell Counting Kit (DOJINDO) was used to assess the proliferation of U14 cells or spleen cells. The optical density of the solution (OD_450_) in each well was measured following addition of 10 μL CCK-8 reagent. For EdU assay, U14 cells were seeded in 24-well plates at a density of 5×10^4^ cells and incubated at 37 °C with 5% CO_2_. The EdU incorporation assay was carried out according to the instruction of the BeyoClickTM EdU-488 assay kit (C0071S, Beyotime). After incubation with 10 μM EdU for 2 h, cells were fixed with 4% paraformaldehyde, permeabilized with 3% triton X-100, and stained with fluorescent dyes. Then samples were analyzed by flow cytometry. For CFSE assay, spleen cells were washed with PBS, resuspended in 1 mL PBS (1×10^6^ cells) and incubated in 1μM CFSE (Thermo Fisher Scientific) for 10 min at 37 °C. The labeled cells were added to the cell culture plate coated with anti-CD3 (1 μg/mL), and simultaneously cultured with anti-CD28 (1 μg/mL) and the indicated concentrations of EPZ015666 for 72 h. Then samples were analyzed by flow cytometry.

### Statistical analysis

Statistical analyses were performed with Prism6 (GraphPad Software). Data were shown as the mean ± SEM and evaluated using a two-tailed Student's T test. P < 0.05 was considered to indicate a statistically significant difference.

## Supplementary Material

Supplementary figures and tables.

## Abbreviations

PRMT5: protein arginine methyltransferase 5
PTM: post-translational modification
MMA: monomethylarginine
ADMA: asymmetric dimethylarginine
SDMA: symmetric dimethylarginine
PD-1: programmed death-1
PD-L1: programmed death-ligand 1
TILs: tumor infiltrating lymphocytes
H3R2me2s: symmetric dimethylation of histone H3 arginine 2
H4R3me2s: symmetric dimethylation of histone H4 arginine 3
H3R8me2s: symmetric dimethylation of histone H3 arginine 8
FBS: fetal bovine serum
PBS: phosphate-buffered saline
shRNA: short hairpin RNA
IHC: immunohistochemistry
ChIP: chromatin immunoprecipitation
