# Supplementary material for: PRMT5 disruption drives antitumor immunity in cervical cancer by reprogramming T cell-mediated response and regulating PD-L1 expression
Source: Theranostics. 2021 Aug 28;11(18):9162–76. doi: 10.7150/thno.59605 (PMC8419032; doi:10.7150/thno.59605)
Supplement: Supplementary file 1 — Supplementary figures and tables. [file thnov11p9162s1.pdf]

Supplementary Materials for

PRMT5 disruption drives antitumor immunity in cervical cancer by reprogramming T cell-mediated response and regulating PD-L1 expression

Yongshuai Jiang[1#], Yuanyang Yuan[1#*], Ming Chen[2], Shengzhe Li[1], Jun Bai[1], Yuanteng Zhang[1], Ying Sun[1], Guojue Wang[1], Haiyan Xu[1], Ziyu Wang[1], Yingxia Zheng[3*], Hong Nie[1*]

[1]Shanghai Institute of Immunology, Department of Immunology and Microbiology, Shanghai Jiao Tong University School of Medicine, Shanghai 200025, China.

[2]Department of Gynecology, Shanghai Ninth People's Hospital, Shanghai Jiao Tong University School of Medicine, Shanghai 200011, China.

[3]Department of Laboratory Medicine, Xin Hua Hospital, Shanghai Jiao Tong University School of Medicine, Shanghai 200092, China.

[#]These authors contribute equally to this article.

* Correspondence should be addressed to Hong Nie (hnie0823@126.com), Yingxia Zheng (combi3230@163.com) and Yuanyang Yuan (sunralmega@126.com)

This file includes:

Figures S1 to S6

Tables S1 to S4

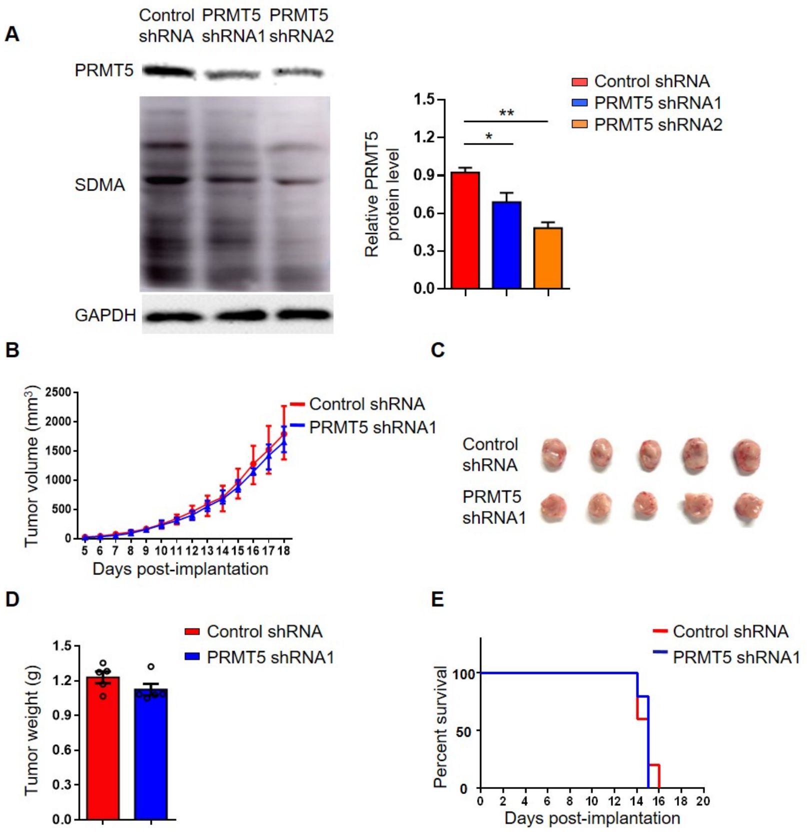

Supplementary Figure 1. PRMT5 disruption had no effect on tumor growth in nude mice. (**A**) PRMT5 expression and SDMA level in control cells and PRMT5 knockdown U14 cells (left panel), and the protein expression of PRMT5 was quantified by ImageJ (right panel). (**B-E**) Control cells and PRMT5 knockdown U14 cells were subcutaneously injected into 6-week-old female nude mice (n = 5 for each group). (**B**) A line graph shows the tumor growth curve of nude mice. Images (**C**) and weight (**D**) of the resected tumor at day 18 after inoculation. (**E**) Survival curve of tumor-bearing nude mice. Data are representative of at least two independent experiments. Values are presented as the mean ± SEM. *P < 0.05 and **P < 0.01.

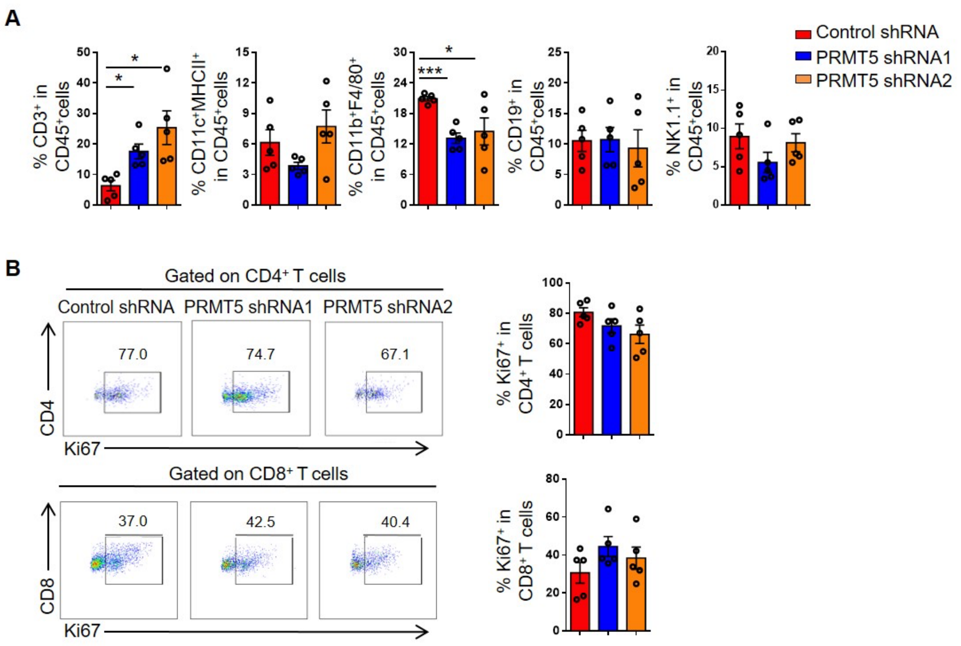

Supplementary Figure 2. PRMT5 deficiency in tumor cells affected the profile of immune cells in the tumor microenvironment. Control cells and PRMT5 knockdown U14 cells were subcutaneously injected into 6-week-old female C57BL/6 mice (n = 5 for each group). Mice were euthanized at day 8 after inoculation. The tumor single cell suspension was prepared and analyzed by flow cytometry. (**A**) Effect of PRMT5 knockdown U14 cells on the population of immune cells in the tumor microenvironment. (**B**) The expression of Ki67 in $CD4^+$ T and $CD8^+$ T cells. Data are representative of two independent experiments. Values are presented as the mean ± SEM. *P < 0.05 and *** P < 0.001.

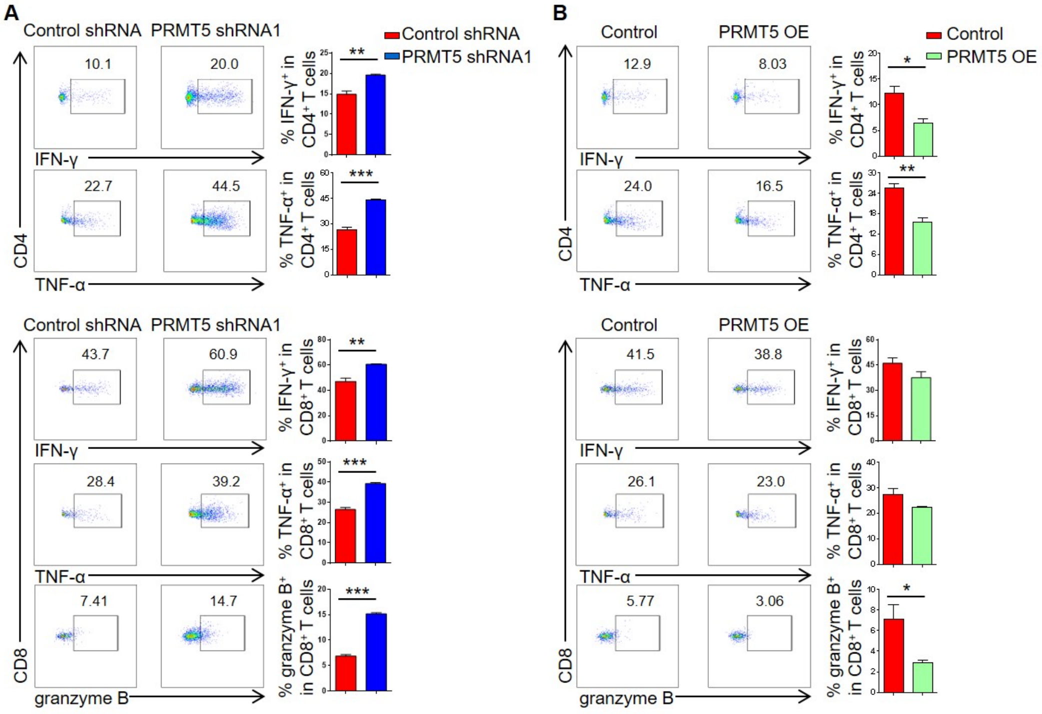

Supplementary Figure 3. Abnormal expression of PRMT5 in cervical cancer cells affected intracellular cytokine expression of T cells in co-culture system. PRMT5 knockdown U14 cells, PRMT5-overexpressing U14 cells or corresponding control group cells were co-cultured with T cells at ratio of 1:10, stimulated with anti-CD3 (2 μg/mL) and anti-CD28 (2 μg/mL) for 48 h. Flow cytometry was used to analyze the expression of IFN-γ, TNF-α and granzyme B in T cells after co-cultured with PRMT5 knockdown U14 cells (**A**) and PRMT5-overexpressing U14 cells (**B**). Data are representative of two independent experiments. Values are presented as the mean ± SEM. *P < 0.05, **P < 0.01 and ***P < 0.001.

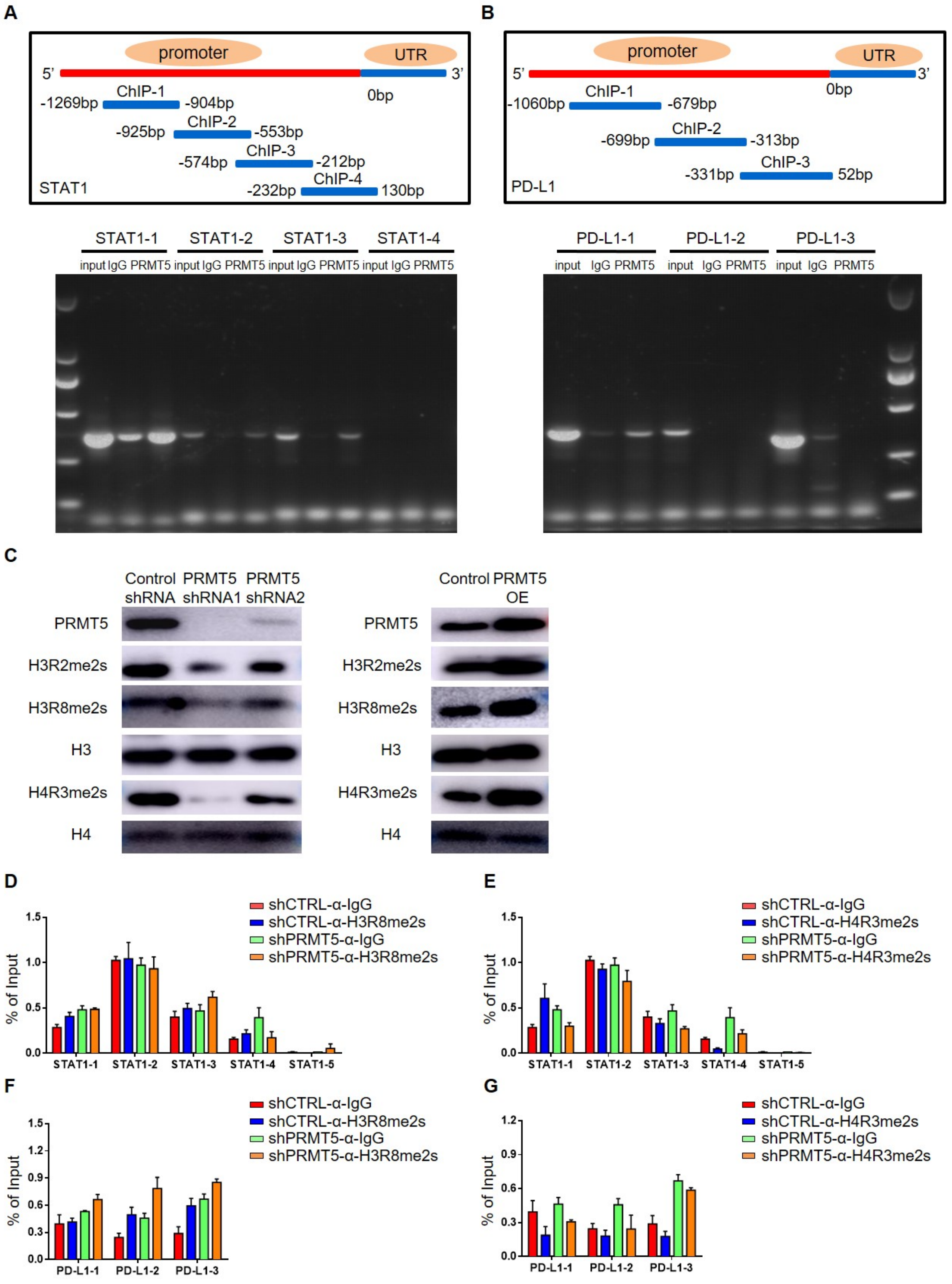

Supplementary Figure 4. PRMT5 regulated STAT1 and PD-L1 transcription and increased H3R2, H3R8 and H4R3 dimethylation level. (**A-B**) Enrichment of PRMT5 or IgG at the STAT1 promoter (**A**) or at the PD-L1 promoter (**B**) was assessed by ChIP PCR. (**C**) The expression of PRMT5, H3R2me2s, H3R8me2s and H4R3me2s in Siha

cells was determined by western blot, and H3 or H4 was served as a loading control. (**D-E**) Enrichment of H3R8me2s, H4R3me2s or IgG at the STAT1 promoter was assessed by ChIP Q-PCR. (**F-G**) Enrichment of H3R8me2s, H4R3me2s or IgG at the PD-L1 promoter was assessed by ChIP Q-PCR. Data are representative of two independent experiments. Values are presented as the mean ± SEM.

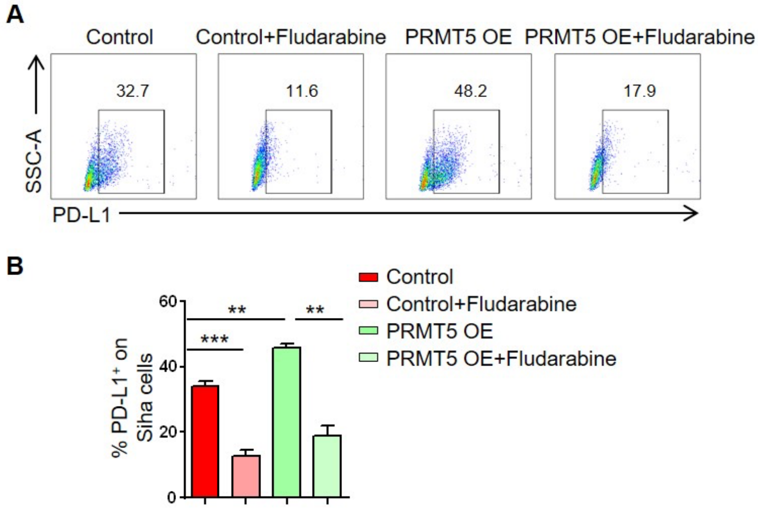

Supplementary Figure 5. A STAT1 inhibitor reduced PD-L1 expression in cervical cancer cells. Control cells and PRMT5-overexpressing Siha cells were simultaneously stimulated with IFN-γ (40 ng/mL) and treated with or without a STAT1 inhibitor (Fludarabine, 5 μM) for 24 h. Flow-cytometric analysis of PD-L1 expression on Siha cells (**A**), and statistical analysis of the percentage of PD-L1 expression (**B**). Data are representative of two independent experiments. Values are presented as the mean ± SEM. **P < 0.01 and *** P < 0.001.

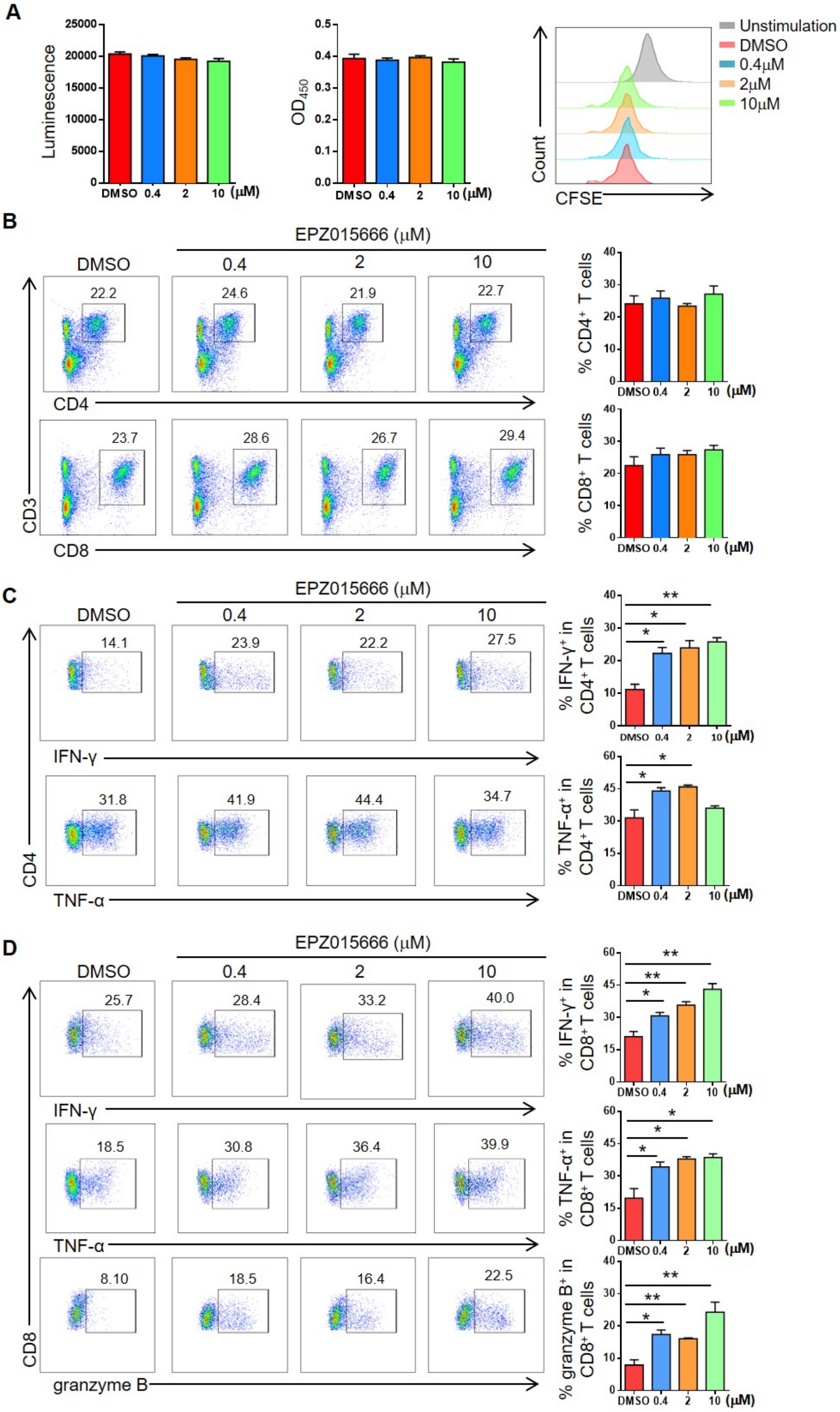

Supplementary Figure 6. EPZ015666 promoted the expression of intracellular cytokines in T cells. C57BL/6 mouse splenocytes were added to the cell culture plate

coated with anti-CD3 (1 μg/mL), and simultaneously cultured with anti-CD28 (1 μg/mL) and the indicated concentrations of EPZ015666 for 72 h (n = 3). (**A**) Effect of EPZ015666 on the viability (left panel) and proliferation (middle and right panel) of mouse splenocytes was analyzed by CellTiter-Glo luminescent assay, CCK-8 Cell Counting assay and CFSE staining, respectively. The percentage of $CD4^+$ T and $CD8^+$ T cells in spleen cells (**B**), the expression of cytokines in $CD4^+$ T cells (**C**) and $CD8^+$ T cells (**D**) were analyzed by flow cytometry. Values are presented as the mean ± SEM. *$P < 0.05$ and **$P < 0.01$.

Supplementary Table 1. Antibodies used in the experiment.

Antibodies	Source	Identifier
Monoclonal Antibody CD45 PerCP-Cyanine5.5	eBioscience	45-0451-82
Rat Anti-Mouse CD45 BV421	BD	563890
Monoclonal Antibody CD3 Alexa Fluor 700	eBioscience	56-0032-82
Monoclonal Antibody CD4 FITC	eBioscience	11-0041-85
Rat Anti-Mouse CD8a PE-CF594	BD	562283
Hamster Anti-Mouse CD279 (PD-1) BV421	BD	562584
Monoclonal Antibody CD274 (PD-L1) PE	eBioscience	12-5982-82
Anti-mouse Antibody CD366 (TIM-3) BV605	Biolegend	119721
Rat Anti-Mouse CD223 (LAG-3) BV711	BD	563179
Monoclonal Antibody IFN-γ PE-Cyanine7	eBioscience	25-7311-82
Monoclonal Antibody TNF-α APC	eBioscience	17-7321-81
Monoclonal Antibody Foxp3 APC	eBioscience	17-5773-82
Monoclonal Antibody granzyme B PE	eBioscience	12-8898-80
Monoclonal Antibody Ki67 FITC	eBioscience	11-5698-82
Fixable Viability Dye eFluor 780	eBioscience	65-0865-18
LIVE/DEAD™ Fixable Blue Dead Cell Stain Kit	Life	L34962

Supplementary Table 2. Primer sequences for quantitative real-time PCR.

hGAPDH	Forward	5'- GGTGGTCTCCTCTGACTTCAACA -3'
	Reverse	5'- GTTGCTGTAGCCAAATTCGTTGT -3'
hPRMT5	Forward	5'- CTGTCTTCCATCCGCGTTTCA -3'
	Reverse	5'- GCAGTAGGTCTGATCGTGTCTG -3'
mβ-actin	Forward	5'- TGTCCACCTTCCAGCAGATGT -3'
	Reverse	5'- AGCTCAGTAACAGTCCGCCTAG -3'
mPRMT5	Forward	5'- CTGAATTGCGTCCCCGAAATA -3'
	Reverse	5'- AGGTTCCTGAATGAACTCCCT -3'
mJAK2	Forward	5'- TTGTGGTATTACGCCTGTGTATC -3'
	Reverse	5'- ATGCCTGGTTGACTCGTCTAT -3'
mSTAT1	Forward	5'- CGGAGTCGGAGGCCCTAAT -3'
	Reverse	5'- ACAGCAGGTGCTTCTTAATGAG -3'
mPD-L1	Forward	5'- GCTCCAAAGGACTTGTACGTG -3'
	Reverse	5'- TGATCTGAAGGGCAGCATTTC -3'

Supplementary Table 3. Primer sequences for ChIP PCR.

STAT1 first site	Forward	5'- AGCCAGCGAAGAGTTGGGTGA -3'
	Reverse	5'- GCCTCCTTCTGCAGTAGATTC -3'
STAT1 second site	Forward	5'- GAATCTACTGCAGAAGGAGGC -3'
	Reverse	5'- CTTCTCCTAAACGCTGTGCTG -3'
STAT1 third site	Forward	5'- CAGCACAGCGTTTAGGAGAAG -3'
	Reverse	5'- CTTGGAGGTCTCTGTAGTAG -3'
STAT1 forth site	Forward	5'- CTACTACAGAGACCTCCAAG -3'
	Reverse	5'- CTCCGCAGACTCTGCGCAGGA -3'
PD-L1 first site	Forward	5'- AACCAATGCAAGGGCTATCTC -3'
	Reverse	5'- GGTCCCTGATATTCTGCCACC -3'
PD-L1 second site	Forward	5'- GGTGGCAGAATATCAGGGACC -3'
	Reverse	5'- CAACAAGCCAACATCTGAACG -3'
PD-L1 third site	Forward	5'- CGTTCAGATGTTGGCTTGTTG -3'
	Reverse	5'- CTCGGGAAGCTGCGCAGAAC -3'

Supplementary Table 4. Primer sequences for ChIP Q-PCR.

STAT1 first site	Forward	5'- CCAGCGAAGAGTTGGGTGAA -3'
	Reverse	5'- TATAAAGCCCTGGTTGCCCG -3'
STAT1 second site	Forward	5'- TTGGACAAAGAGATCGGGCAA -3'
	Reverse	5'- TGCCTCACTTCAGCCCTTAT -3'
STAT1 third site	Forward	5'- AAGTGAGGCAGCCATTCGGG -3'
	Reverse	5'- TGCAGTGAGCTCTACAAACT -3'
STAT1 forth site	Forward	5'- CACTGCACATACAAGTGGAG -3'
	Reverse	5'- CCTGCCTCTGGCATTCTTTC -3'
STAT1 fifth site	Forward	5'- AGGCAGGAAAAAGCAAGAAG -3'
	Reverse	5'- CTTGGAGGTCTCTGTAGTAG -3'
PD-L1 first site	Forward	5'- AACCAATGCAAGGGCTATCTC -3'
	Reverse	5'- GTGCCTGTGTGCTCCCTTTTC -3'
PD-L1 second site	Forward	5'- AAGGGAGCACACAGGCACGG -3'
	Reverse	5'- GGGCCCAAGATGACAGACGATG -3'
PD-L1 third site	Forward	5'- GTCTGTCATCTTGGGCCCATTC -3'
	Reverse	5'- CAGGGTCCCTGATATTCTGCC -3'
